# Umbilical Venous Catheter Update: A Narrative Review Including Ultrasound and Training

**DOI:** 10.3389/fped.2021.774705

**Published:** 2022-01-31

**Authors:** Vito D'Andrea, Giorgia Prontera, Serena Antonia Rubortone, Lucilla Pezza, Giovanni Pinna, Giovanni Barone, Mauro Pittiruti, Giovanni Vento

**Affiliations:** ^1^Division of Neonatology, Department of Woman and Child Health and Public Health, University Hospital Fondazione Policlinico Gemelli Istituto di Ricovero e Cura a Carattere Scientifico (IRCCS), Rome, Italy; ^2^Neonatal Intensive Care Unit, Infermi Hospital, Rimini, Italy; ^3^Department of Surgery, University Hospital Fondazione Policlinico Gemelli Istituto di Ricovero e Cura a Carattere Scientifico (IRCCS), Rome, Italy

**Keywords:** umbilical venous catheters, POCUS, CRBSI (catheter-related bloodstream infection), catheters and catheterization complications, catheters and catheterization, technology, training

## Abstract

The umbilical venous catheter (UVC) is one of the most commonly used central lines in neonates. It can be easily inserted soon after birth providing stable intravenous access in infants requiring advanced resuscitation in the delivery room or needing medications, fluids, and parenteral nutrition during the 1st days of life. Resident training is crucial for UVC placement. The use of simulators allows trainees to gain practical experience and confidence in performing the procedure without risks for patients. UVCs are easy to insert, however when the procedure is performed without the use of ultrasound, there is a quite high risk, up to 40%, of non-central position. Ultrasound-guided UVC tip location is a simple and learnable technique and therefore should be widespread among all physicians. The feasibility of targeted training on the use of point-of-care ultrasound (POCUS) for UVC placement in the neonatal intensive care unit (NICU) among neonatal medical staff has been demonstrated. Conversely, UVC-related complications are very common and can sometimes be life-threatening. Despite UVCs being used by neonatologists for over 60 years, there are still no standard guidelines for assessment or monitoring of tip location, securement, management, or dwell time. This review article is an overview of the current knowledge and evidence available in the literature about UVCs. Our aim is to provide precise and updated recommendations on the use of this central line.

## Introduction

The umbilical venous catheter (UVC) is one of the most frequently used central venous access devices in the neonatal period. It can be easily placed and it is extremely useful for preterm and/or for critically ill infants requiring frequent blood sampling and intravenous administration of fluids, medications, and parenteral nutrition. The correct tip location is at the junction between the inferior vena cava (IVC) and the right atrium (RA), which can be reached after entering the umbilical vein and passing through the ductus venosus (DV) (1–5). This position is considered to be associated with the lowest incidence of complications.

For decades, thoracoabdominal radiograph (TAR) has been used to assess the position of the catheter's tip, but over the past few years ultrasonography has been suggested as the gold standard technique since it is safe, fast, and more accurate. Ultrasound is also ideal for daily evaluation of tip location, since tip migration may occur frequently (6–13). Indeed, UVC-related complications can be very severe, so it is important to check the catheter's tip over time and to keep in mind the possible implications of this central line.

Although neonatologists have been using UVCs for more than 60 years, the standard of care in the management of such a device is still a matter of debate. For instance, the most appropriate indwelling time and the best method for securement are still not well-defined.

The goal of this narrative review is to offer a practice-oriented overview on UVCs, looking at the more advanced technologies, some of which is already evidence-based, that might improve clinical outcomes.

## Indications and Indwelling Time

UVC is frequently used in the neonatal intensive care unit (NICU) because it provides safe vascular access immediately after birth in high-risk newborns. UVCs are typically used for intravenous administration of parenteral nutrition and drugs, for blood sampling, and for blood transfusions (14).

At the time of insertion, it is often not easy to predict the clinical course of the newborn, so there is a significant risk of overusing this device, especially in preterm infants.

In a quality improvement document aiming at reducing unnecessary placement of UVCs, Shahid et al. (15) developed a consensus guideline providing indications for UVC placement on the basis of gestational age (GA), severity of illness, and difficulty of establishing peripheral intravenous vascular (PIV) access. They recommended the use of UVC in all preterm infants ≤28 weeks, and in newborns ≥29 weeks mechanically ventilated or with FiO_2_ >40% on continuous positive airway pressure and/or hemodynamically unstable and/or needing inotropes or fluids bolus, or with difficulty establishing PIV access.

According to the Michigan Appropriateness Guide for Intravenous Catheters in pediatrics recommendations (16), the use of UVC in term neonates is influenced by the infusate characteristics, expected duration of therapy, and age of the neonate. In this document, the insertion of UVC is considered appropriate up to day 5 after birth for non-peripherally compatible infusions. The Centers for Disease Control and Prevention Hospital Infection Control Practices Advisory Committee recommends that UVCs should be removed as soon as possible, but they can be used for up to 14 days if absolutely needed and if managed aseptically (17).

In 2012, Butler-O'Hara et al. (18) compared retrospectively UVCs with dwell time ≤7 vs. >7 days. The paper proved that the rate of central line-associated bloodstream infection (CLABSI) was 1/1,000 catheter days in the ≤7 days UVC group vs. 4/1,000 catheter days in the >7 days UVC group (*P* < 0.001). Other authors (19) suggested that UVCs should be removed earlier, before day 4, and replaced by another central venous catheter. In a multicenter retrospective study, the authors demonstrated that the risk of CLABSI is proportional to the dwell time of the UVC. In a systematic review, Keir et al. (20) concluded that if central access is required beyond 5–7 days, the UVC should be removed and replaced with another central line. INS 2021 guidelines suggest limiting UVC dwell time to 7–10 days and UVC removal at 4 days followed by insertion of a PICC for continued infusion as an infection prevention strategy (5).

## Choice of the Device

Polyurethane catheters are preferred over polyethylene and polyvinyl catheters, since they are less prone to bacterial colonization. Polyurethane UVCs that release antimicrobially active silver ions have recently become available in the market. These catheters are potentially associated with a reduced risk of CLABSI, since they decrease both endoluminal and extraluminal colonization (21). A single randomized controlled study (22) has demonstrated that these silver-impregnated UVCs are effective in decreasing the risk of catheter-related bloodstream infections (CRBSI) in preterm infants with gestational age < 30 weeks. Their use has also been recommended by SHEA guidelines in 2014 for prevention of CRBSI in preterm infants (23). However, looking at the data from the recent Cochrane study published in 2015 (24), it seems reasonable to recommend these catheters only for preterm infants and in the neonatal unit with a policy of long dwell time for UVC (more than 7 days). In fact, the Kaplan-Meier estimates clearly show that the differences in the risk of CRBSI between conventional UVCs and antimicrobial impregnated ones becomes clinically relevant for dwell time longer than 7 days.

As regards the caliber of the UVC, 3.5 Fr catheters are usually recommended for infants weighing <3.5 kg and 5 Fr catheters for infants weighing more than 3.5 kg. Double and triple-lumen catheters are available if simultaneous administration of incompatible solutions is anticipated. The use of multi-lumen catheters may reduce the need for additional PIVs and is recommended in low birth weight infants (25).

## Insertion of the UVC Including New Technologies

Several studies have tried to define the best length estimation technique during UVC insertion. In 1966, Dunn published a graph reference, mainly based on the distance between shoulder and umbilicus (26). Twenty years later, Shukla and Ferrara came up with a formula based on body weight (BW) (27), which was further modified by Verheij et al. (28). Another retrospective study proposed the use of distance from the umbilicus to the mid-xiphoid (29). One more formula has been proposed by Gupta (30). Table 1 summarizes all these different methods. At present, there is no formula, nomogram, or measurement, that can be universally and effectively applied to infants of different BWs and Gas (13, 31, 32). Though, the methods most commonly used for estimation of catheter length are the Dunn formula and the Shukla formula. The Shukla formula has the highest rate of either correct or high position. The Dunn formula, on the other side, seems to perform quite poorly.

**Table 1:** Different methods to estimate a correct UVC insertion length.

**References**	**Study population**	**Results**
Dunn (26)	50 UV (at necropsy) BW 680–4,027 g, EG 26–44 weeks	Shoulder-umbilicus distance
Shukla and Ferrara (27)	39 UV and 4 UA, BW 2,037 ± 1,077 g 10 UV, BW 2,260 ± 1,144 g	Lenght (cm) = [(3 * BW in Kg + 9) / 2 +1]
Verheij et al. (28)	143 UV using the Shukla formula 125 UV using the revised formula	Lenght (cm) = [(3 * BW in Kg + 9) / 2]
Vali et al. (29)	82 UV and 55 UA BW 1,311± 888 g, EG 23–27^+6^ weeks	Umbilicus-mid-xiphoid-bed distance
Gupta et al. (30)	170 UV, BW 490 ± 4,800 g, EG 24–41 weeks 125 UA, BW 490 ± 4,800 g, EG 24–41 weeks	Lenght (cm) = Umbilical-Nipple length – 1 cm

*The Shukla formula had the highest rate of either correct or high position. The Dunn formula performed quite poorly*.

In emergency use, the UVC tip should be located only at a short distance, 2–4 cm, at the beginning of the umbilical vein to the point at which blood can be freely aspirated. Indeed, if the catheter is inserted further, there is risk of hepatic injury caused by infusing medications directly into the liver (33, 34).

**Figure 1:**
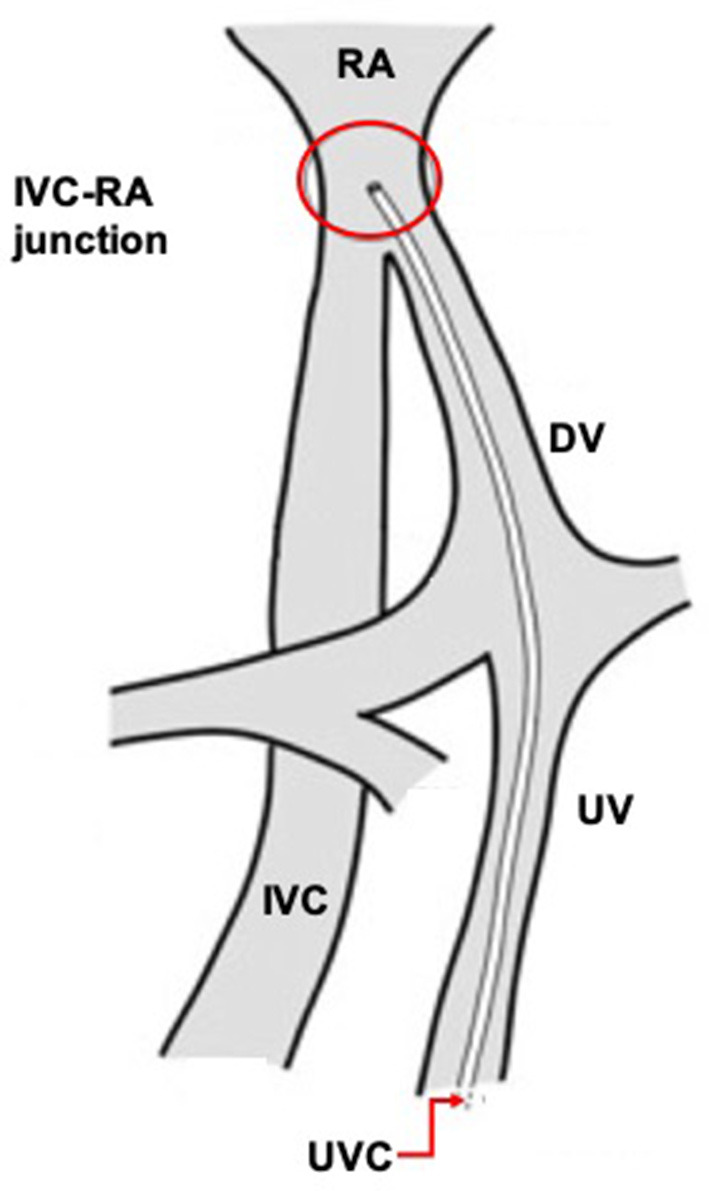
Correct umbilical venous catheter position.

At the time of insertion, the UVC first enters the umbilical vein, then the medial part of the left portal vein and the DV, eventually reaching the junction of IVC and RA (Figure 1). Unfortunately, the UVC might take some collateral route into the portal system and be accidentally located in an intrahepatic branch of the portal vein.

Observational studies showed that some maneuvers could facilitate UVC passage through the DV. Pennaforte et al. (35) have recommended the manual mobilization of the liver during UVC insertion. Other authors (36) have suggested placing the infant on the right side during UVC insertion, since this position might reduce the risk of tip progression into the portal venous circulation. Another maneuver suggested is the compression of the upper abdomen near the portal sinus of the liver, to align the umbilical vein and DV, under ultrasound guidance (37). The Accreditation Council for Graduate Medical Education (ACGME) expects pediatric residents to become competent in performing certain procedures during general pediatric practice, including umbilical line placement (38). Several authors have studied the most effective method for adequate training of pediatric residents in the placement of UVCs (39, 40). Haviland et al. conducted a study in which nine post-graduate 1st year residents completed a pre-training survey and simulation, and eight residents completed a post-training survey and simulation. The residents demonstrated an increase in objectively measured competence and self-rated confidence, despite lacking opportunities to perform the procedure on live neonates (41). Overall, simulation is an important component of residency training, especially since it allows residents to gain hands-on experience without risks to the patients.

**Figure 2:**
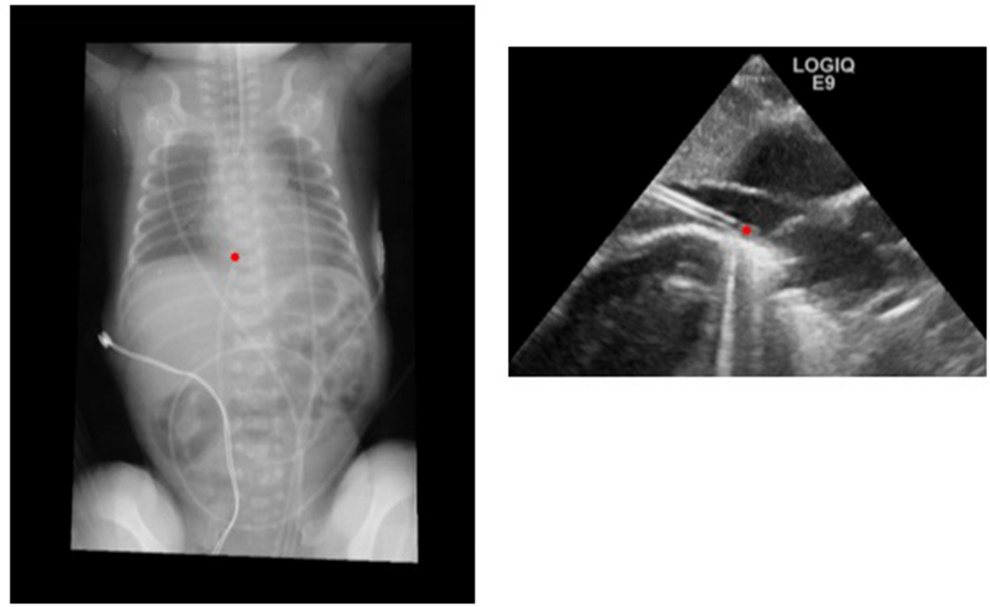
Comparison of radiographic and ultrasound images of a correctly positioned UVC.

The newest and most interesting development in UVC insertion is indeed the introduction of ultrasound-based methods for tip navigation and tip location. The use of point-of-care ultrasound (POCUS) to visualize umbilical catheters during the procedure is rapidly spreading among neonatologists (11, 12, 42–44) (Figure 2).

In 2013, Pulickal et al. conducted a prospective observational study in which real-time ultrasonography was performed by a trained cohort of pediatric house staff physicians during UVC insertion (10). Many other studies confirmed the superiority and higher accuracy of intraprocedural ultrasound over post-procedural radiologic assessment (8, 45, 46). Chest X-ray is inaccurate as X-rays do not allow the visualization of the veins. Therefore, clinicians must infer the location of the tip of the catheters on the basis of other radiological landmarks such as the vertebral bodies, cardiac silhouette, and the diaphragmatic contour. On the other side, ultrasound allows a clear view of the vein and of the catheter itself. In the studies quoted, 20–25% of the catheters that were judged to be in a correct position on the chest X-ray were readjusted after the ultrasound was performed.

Despite all this evidence, POCUS is not universally used in NICU for this purpose, possibly because it needs specific and adequate training of the neonatal medical staff.

In a recent experimental study, Kaee et al. investigated the learning curve for targeted ultrasound evaluation of UVC placement in physicians with low experience in ultrasound use in a piglet model. After a short eLearning module and a few practical sessions, trainees were able to detect the catheter's tip with accuracy and high self-confidence score within 10 min (47). A recent pre/post intervention study demonstrated the feasibility of targeted training on the use of POCUS for UVC placement in NICU among neonatal medical staff. The technique was easily teachable, increased the number of UVCs correctly positioned, and reduced the number of line manipulations and chest X-rays (48).

Similarly, ultrasound performed by neonatal residents and neonatal nurse practitioners has good results in terms of diagnostic accuracy, after adequate training (49, 50).

Realtime ultrasound for tip navigation and tip location of UVC is accurate, safe, and cost-effective. It is reasonable to predict that real-time ultrasound will shortly become a new standard of care with the support of standardized protocols defining the proper technique (42).

## Securement

Four different methods of UVC securement have been described (51). In the “anchoring” technique, the UVC is sandwiched between two small pieces of tape then reinforced by stitching from the tape through the umbilical cord or skin on both sides. In the technique of “bridging,” two thin pieces of tape are placed on each side of the umbilicus in a T-shape and secured to the abdomen, another piece of tape is then bridged between the two T-pieces that holds the catheter in place. Another method uses a DuoDERM patch which is cut to fit the baby's chest and the umbilical catheter is rolled up and secured to it by a piece of transparent dressing. In the last technique, the UVC holder adheres to the skin with hydrocolloid gel and two flaps rise up from the base and open and close multiple times for adjustments. In many centers, the UVC is secured only using sutures, without tape or other adhesive materials, as described in McDonald's Atlas of Procedures in Neonatology (14). In conclusion, in the current literature there are no data that support one method over another, so the choice is made according to local guidelines, medical skills, and to the infant's conditions. A randomized controlled trial is in progress in our neonatal unit and proposes the use of cyanoacrylate glue applied to the umbilical stump to reinforce the securing suture and ensure a better stabilization (52). Probably a strategic choice could include the use of a sutureless device, the use of cyanoacrylate glue in order to provide immediate hemostasis and to prevent dislodgement, and the application of a transparent semipermeable dressing with a high level of moisture transmission rate.

## Care

A high standard level in the care of the UVC in NICU is crucial for the prevention of infective and mechanical complications. Particularly good clinical practice should include daily assessment and care of the exit site, proper securement, appropriate skin antisepsis, bundles for flush and lock, and administration set management.

UVC is a central line and therefore should be managed accordingly. We do recommend a thorough reading of the recent INS guidelines published in 2021 (5), that summarize the standard of care regarding administration set changes, the proper use of a needleless connector and port protector, and the appropriate policy of flushing and locking using normal saline with a prefilled syringe.

Currently there is no universal agreement on the appropriate skin antiseptic. However, the single use applicator containing 2% chlorhexidine in 70% isopropyl alcohol seems to be safe even in very low birth weight infants.

The main outstanding issue regarding the management of the UVC remains the proper securement and the care of the exit site. Unfortunately, no guideline is available yet about this topic and several approaches have been proposed in the literature.

## Post-Procedural X-Ray

The most common method to confirm tip location is still the anteroposterior TAR. Two methods of radiograph interpretation have been described in literature. In the “cardiac silhouette” method, the position of the cavoatrial junction (the target zone) is estimated by extrapolating the curve of the right atrial border medially to its intersection with the IVC (which is best determined when the UVC passes through the IVC) or the right border of the vertebral bodies (if the UVC does not pass through the IVC). In the “vertebral body” method, the tip is defined as “well-positioned” when located at the level of T8–T9, “high” if above T8, and “low” if below T9 (53, 54). The “cardiac silhouette” method is more accurate than the “vertebral body” method (54).

Radiological methods of tip location should be discouraged. As compared to intraprocedural ultrasound (42), post-procedural radiological assessment of UVC position implies an inevitable delay in starting the infusions, is more invasive, more difficult from the logistic point of view, less accurate and less cost-effective, and—last but not least—it is less safe. In fact, in a retrospective analysis of 215 premature infants, Scott et al. (55) found that 12.1% of the infants with a GA <33 weeks received more than the maximum recommended ionizing radiation exposure (1,000 mSv) during their NICU stay and that central lines placement accounted for 19.2% of the total radiation exposure.

## Migration

Migration of UVC after insertion has been widely documented. Migration might occur in 50%, 63%, or even in 90% of cases (45, 56, 57). It is commonly attributed to the drying of Wharton jelly and the secondary shortening of the umbilical cord.

A prospective cohort study quantified the direction and the magnitude of catheter's tip migration (58). The authors described an inward migration pattern during the first 48 h after UVC placement, followed later by an outward migration. The inward migration was explained by cord stump contraction over time together with an increase in lung volume (due to the increase in the functional residual capacity, e.g., following surfactant administration), the outward migration instead was likely to result from gradual distension of the abdomen as the bowel filled with gas (58). About half the infants included in the studies experienced migration, mainly inward within 24–48 h from the catheter insertion (48, 59, 60).

The risk of tip migration is a strong reason for adopting ultrasound-based tip location, which can be safely repeated over time, even daily (42).

## Infection

The use of any central line, including UVC, is always associated with the risk of infection. Prevention of CLABSI is fundamental as both neurodevelopmental and growth outcomes are negatively affected in infants with postnatally acquired infections (61). The most common microorganisms are coagulase-negative staphylococcus (CONS) followed by Gram-negative bacilli and fungi.

The use of UVCs has been associated with colonization in 22–59% of cases and with CRBSI in 3–20% of cases. This huge variability is mainly related to the mean dwell time throughout the different studies. CRBSI was defined by a culture of a peripheral, percutaneously obtained blood sample that was positive for the same organism found to be colonizing the UVC hub or tip (i.e., concordant colonization of the catheter hub or tip). Colonization means the presence of ≥15 CFU of a single organism per catheter if not accompanied by a laboratory-confirmed blood stream infection of the patient. In patients requiring long-term central venous access, CRBSI is reduced by nearly half after the institution of a dedicated vascular access team in NICU (62). Likewise, other authors showed that line bundles and dedicated line care teams decrease the risk of CRBSI and CLABSI (17, 63, 64).

## Non-infective Complications

UVCs are also potentially associated with severe non-infective complications (65).

UVC with the tip in the right atrium might be associated with cardiac complications. Fourteen cases of cardiac arrhythmias associated with UVCs have been reported in literature, mainly atrial flutter and paroxysmal supraventricular tachycardia (66). Cardiac tamponade caused by UVC is rare (0.5–2% incidence) but life-threatening, and it can occur even when the catheter is properly positioned. The pathogenesis is most likely due to erosion of the vascular or cardiac wall by the catheter leading to perforation. The hyperosmolar fluid infused through the line diffuses into the pericardial space (67, 68).

UVCs with the tip in the hepatic vessels or the portal system are associated with high risk of extravasation and consequent parenchymal injuries or portal thrombosis. Hepatic complications due to UVC malposition are common and widely described in numerous case reports (69, 70). Liver ultrasound is the gold standard to diagnose hepatic complications and to follow their evolution over time. Air in the portal venous system is the most frequent finding (20.1%), followed by parenchymal lesions (7.4%) and left portal venous thrombosis (6.1%) (69). The UVC tip can also cause a structural injury, including hepatic hematoma, due to a direct erosion or laceration of an intrahepatic vascular wall. Parenteral nutrition, inotropes, and hypertonic solutions can cause endothelial damage and leakage to the liver parenchyma, resulting in abscess, liver necrosis, or parenchymal tears (71–74). After disruption of the liver capsule and effusion of the collection into the peritoneum, ascites might be seen as a further complication of a malpositioned UVC (73, 75, 76).

Central venous umbilical catheterization is reported to be the most common cause of neonatal thrombosis (77). Catheter-related portal vein thrombosis (PVT) has been described as a rare event but is increasingly recognized thanks to the increased use of ultrasound evaluations when the UVC is in place and after its removal. Reported incidence varies from 2.2 to 43% due to differences in study design and methodology. UVC can cause thrombosis with different mechanisms: direct damage to vessel walls, disrupted blood flow, infusion of substances damaging endothelial cells, and introduction of a foreign thrombogenic surface (77, 78).

UVCs have also been associated with the development of intracardiac thrombosis, pulmonary embolism, and renal vein thrombosis due to tip malposition (79).

A recent prospective cohort study found a significant association between UVC malposition in the portal system or in the DV (low lying line) and an increased incidence of NEC in preterm infants (79, 80). In an experimental model, the authors demonstrated that transient portal hypertension might result from closure of the DV shortly after birth causing intestinal injury. This suggests the potential role of DV occlusion caused by UVC in the development of NEC in premature infants (81).

## Future Medical Education of UVC

UVC placement is a well-established procedure in NICU but nowadays we can take a glance into the future using some promising tools. Certainly, the use of the umbilical cord model opens up a new perspective for teaching catheter placement because it allows the physician, especially trainees, to gain proficiency and confidence with the procedure without risks for the patients. Sawyer et al. described how to create a real human umbilical cord simulator for emergency UVC placement training. The model used a fresh (rather than frozen) human umbilical cord, a newborn simulator with a hole in the abdomen for the umbilical cord, a baby bottle nipple and closure ring, a rubber exam glove, and an umbilical clamp or Kelly clamp (39). They then conducted a randomized crossover trial of senior pediatric residents randomized in groups each with a different UVC simulator, either a real cord (RC) or simulated cord (SC). The two groups then switched their simulators. The authors concluded that the time to place a UVC was slower in the residents using real cords as compared with the simulated cords, however, there was no difference in the time taken to place an eUVC in the group that worked with the simulated cords first (40). In our NICU the training program includes:

— theoretical instruction by an expert to first-year residents.
— practical exercises once a week on the simulator.
— after 3 months, evaluation of sequence procedure on simulator.
— the placement on the small patient is supervised by a senior resident at least 3 years old.
— after 20 UVC placements with supervision and the final evaluation, the resident is independent.

The use of POCUS for tip navigation and location during UVC placement is feasible in any setting, easy to teach, and easy to learn. It reduces catheter malpositioning and associated complications; it also allows safe administration of total parenteral nutrition reducing the waiting time that is usually necessary when X-rays are used. The subcostal longitudinal view is the most commonly used acoustic window, which allows staff to visualize the IVC and the RA, which are the targets. The tip is followed until it reaches the target zone. A small flush of normal saline (0.5–1 ml) can improve the visualization of the tip (42). Finally, the use of a wireless ultrasound probe connected with a smartphone or tablet could be very beneficial in the UVC's tip location. It is easy to use for any medical and paramedical operator, it can be used in emergencies, in training, and above all in the diagnostic management of the isolated patients (82).

Current technological advancements are providing new modalities for simulation-based training. One such promising approach is Augmented Reality (AR) which is created by combining real and virtual data with real-time interactivity and three-dimensional registration. A recent review of available AR applications revealed that although the use of this technology is gaining interest, data are still lacking to support its overall applicability and effectiveness (83). Categories of current AR applications were limited to the disciplines of laparoscopy, neurosurgery, and echocardiography. Currently there is no such paper in the field of venous access in newborns but AR could soon play a large role in neonatal vascular training.

## Conclusion

UVC provides vascular access immediately after birth in preterm and/or critically ill infants who require fluid resuscitation, intravenous medications, and parenteral nutrition; it can be inserted painlessly and quickly. Nevertheless, an indwelling UVC is associated with many complications. The most effective way to minimize UVC-related adverse events is not to have an UVC in place. As a consequence, the need for UVC placement must be carefully evaluated by choosing selected patients with specific indications. Once in place, catheter management is crucial: line bundles and dedicated line care teams have been shown to decrease the risk of catheter-related infections and the use of POCUS has been proved to be an invaluable tool to prevent complications related to UVC malposition and migration.

Given the high risk of complications and according to the current available evidence, UVCs should be used with caution and an early planned removal is recommended if the clinical indication is no longer present. This strategy is crucial to reduce the incidence of infection and associated morbidity and mortality. If long-term (>4 days) central venous access is required, replacement of the UVC with an epicutaneo-caval catheter (ECC) or with ultrasound-guided central venous access might be beneficial (Table 2).

**Table 2:** Correct steps in UVC management.

1	Choice of selected patients with precise indications
2	Use of silver-impregnated catheters if plan to keep the device *in situ* for more than 7 days in preterm infants (recommended to prevent CRBSI)
3	Line bundles and dedicated line care teams (recommended to prevent CRBSI)
4	Use of point of care ultrasound to visualize catheter tip location during insertion and detect late catheter migration (to prevent mechanical complications)
5	Early removal of UVC (within 4 days)

A standardized program for residents on insertion and ultrasound visualization should be implemented in the future. Synthetic and real simulators should be used weekly as has been done for other types of procedures (intubation). Ultrasound training for UVC tip location and tip navigation is quick and can be learned easily. Wireless ultrasound technology can be used during training and in special settings (isolated neonates or delivery room or emergency room).

## Author Contributions

VD'A, GP, and SR contributed to conception and design of the study. LP organized the database. GB and GP performed the analysis. GP wrote the first draft of the manuscript. VD'A, MP, and GV wrote sections of the manuscript. All authors contributed to manuscript revision, read, and approved the submitted version.

## Conflict of Interest

The authors declare that the research was conducted in the absence of any commercial or financial relationships that could be construed as a potential conflict of interest.

## Publisher's Note

All claims expressed in this article are solely those of the authors and do not necessarily represent those of their affiliated organizations, or those of the publisher, the editors and the reviewers. Any product that may be evaluated in this article, or claim that may be made by its manufacturer, is not guaranteed or endorsed by the publisher.
